# A Libel on the Profession

**Published:** 1894-05

**Authors:** 


### A LIBEL ON THE PROFESSION.

After the adjournment of the State Medical Association a reporter on the *Austin Daily Statesman* published the following scurrilous article, entitled "The Reason Why Doctors Object to Patent Medicines, as Explained by One of Them:"

At the meeting of the doctors' convention here last week the Man of the World chanced to hear a most remarkable assertion made by one of the doctors. The gentleman in question said: "No doctor who has any consideration for his calling will prescribe a patent medicine compound for a patient. If he is a doctor at all he will write a prescription containing the same ingredients. By this means the druggist is enabled to make his percentage on the prescription and the doctor gets a rebate. Now, if the doctor prescribes a patent medicine, the druggist only gets a small margin of profit and the doctor gets nothing at all. So in consideration of this fact, if for no other, a doctor should never prescribe a patent medicine." Now, that is certainly the greatest exposure of quackery and deceit ever exhibited. The Man of the World is glad to note that a delegate and not an Austin physician was the author of the above quoted speech. Upon investigation at a drug store by the writer it was ascertained that not infrequently physicians write out prescriptions for an ounce or so of patent medicine stuff, of course, concealing that fact from the patient, though the druggist readily knows black from white.

"What do you do in such cases?" was asked.

"We simply open one of the bottes of patent medicines we have on the shelf, fill a small phial full of it over the prescription counter and charge the patient $1 for it, and put the big bottle of patent medicine back for just such another sucker. If it hadn't been for that worthy doctor's prescription the man could have got the whole bottle of patent medicine for 50 cents, containing five times as much as that he paid one dollar for on prescription. But in case he was allowed to buy the patent medicine the doctor would have gotten no rebate on the prescription, so there you are in a nutshell."

Such detailed facts as the above are simply appalling. To think that for a mere pittance they would get out of a 50 cents prescription these doctors would go to work and make a poor man pay out $2.50 for five phials of a medicine, that, had he been permitted to pay for in the bottle at first, would only have cost him 50 cents. The mere thought of such a thing is disgusting, and the Man of the World never felt such supreme contempt for any man as he did for the gentleman who made that speech on the convention floor. The speech conveyed only too plainly the information that the doctor was out for spoils—none of the milk of human kindness flowed in his veins. Every 50 cents prescription made a rebate for him, and he was going to have it, come what would.

The Man of the World would suggest that if the able doctors can bring no more forcible argument against the patent medicines than the one given by this doctor in open convention they had better profit by the saying—"Silence is golden."

A number of Austin physicians met and passed the following resolutions, which were also published in the *Statesman:*

At a meeting of representative physicians of Austin, held May 4th, for the purpose of considering and acting on a certain article which recently appeared in the local editorial columns of the *Austin Daily Statesman*, reflecting upon the medical profession of this city, and the entire State, the following resolutions were unanimously adopted and ordered published in the *Statesman:*

WHEREAS, There recently appeared in the local editorial or reportorial columns of the *Statesman* an article headed "The Reason Why Doctors Object to Patent Medicines, as Explained by One of Them," in which article it is asserted that the writer had heard a delegate on the floor of the recent Medical Convention in this city state in substance, "That by writing a prescription for the same ingredients of which a patent medicine is composed, the druggist makes a larger profit and gives the doctor a rebate;" and further, that he, the said writer, in a conversation with an Austin druggist, was informed by said druggist that "it is a common practice for doctors to prescribe a small quantity of a patent medicine and have it put up as a prescription, the druggist charging a much larger price for this part of the bottle thus dispensed than the whole bottle would be sold for; and by that means he made a larger profit, and gave the doctor a rebate." Or, in other words, that this druggist stated, in effect, that doctors and druggists are in collusion to rob their patrons.

*Resolved*, That the statement that a delegate on the floor of the recent medical convention in this city, at any time during its sessions, made any such statement, is false and libelous upon the medical profession; that members of the Austin profession who were present and heard all the discussion on the subject of patent medicines, assert positively that no such statement was made by any member or delegate at any time, and that no language was used that could, by any honest interpretation, be so construed.

*Resolved*, That the statement, by whomsoever made, that any reputable physician in this city is, or has been guilty of any such collusion as is in said article attributed to them, is false and libelous; and we assert that no reputable physician asks, expects or would receive from his druggist any rebate or percentage on his prescriptions.

*Resolved*, That the entire article is a misrepresentation of facts, and an unmerited insult to the entire medical fraternity of this city and State.

J. W. McLaughlin, M. D., President Texas State Medical Association; T. J. Bennett, M. D., President Austin District Medical Society; Q. C. Smith, M. D.; S. E. Hudson, M. D., President Travis County Medical Society; R. M. Swearingen, M. D.; F. E. Daniel, M. D., Editor TEXAS MEDICAL JOURNAL; A. N. Denton, M. D.; E. V. Hamilton, M. D.; T. J. Tyner, M. D.

The young man of the *Statesman* who wrote the article insists that a delegate *did* make the statement attributed to him, and that it was made *Thursday night*. Now, the editor of the JOURNAL was present on Thursday night, as were Dr. McLaughlin, the new President (who had just been inducted into office), Dr. Q. C. Smith and several others, and we assert positively, and are sustained in the assertion by the gentlemen named—that the subject of patent medicines *did not come up, in any form, that night; and not a word of discussion was had on it.* After the induction into office of the officers elect, memorial service was held; after that, Dr. Osborne introduced a resolution condemning contract practice and physicians bidding for practice, and after discussion had begun on this resolution, President Sears promptly ruled it out of order, and explained that the night meeting was called for a special purpose.

Now—the young man of the *Statesman* has gotten things mixed. On Friday morning the subject of patent medicines was discussed. It came up in the resolution of Dr. Cerna, condemning medical journals for publishing certain advertisements; and

Dr. Poyner, of Bartlet, Texas, described the difference between patent medicines and proprietory medicines; and he is the only man who did dilate on the subject. The young man is evidently referring to Dr. Poyner, and has misquoted him badly. We wrote Dr. Poyner, enclosing the article from the *Statesman*, and expressed the opinion that it was his remarks the reporter claimed to be reporting, and asked the doctor to let us know exactly what he did say. Here is Dr. Poyner's reply:

I cannot give the words used in discussing the resolution, but my recollection is very clear as to the thought in my mind. That was, that the line should be clearly drawn between a patent and a secret medicine. That if a man saw proper to take out a patent on any preparation, or name thereof, that was his clear right by the law and as a business proposition. That the law of copyright and patentright stood on the very same basis. That when a thing was patented it was no longer secret, but that the records were open to the inspection of the world. That it would have been well, better for all, if the charlatans who invented the obstetric forceps had taken out a patent, instead of concealing their invention and using it for their gain exclusively. That such coal tar products as antipyrine, phenacetine, and antifebrin, were not secret as to composition, but the name only was proprietary. That I had never prescribed antikamnia, because I did not know its exact composition, though probably it was nearly identical with the anodyne tablets made by Sharp & Dohme, formula on bottle. I alluded to the fondness for secrecy and voodooism among the laity. Also the ridiculous attitude that a physician would occupy if put on the stand in a suit for malpractice and asked for the composition of his prescription, supposing he had prescribed antikamnia, etc.—secret preparations.

This is about all on the Cerna resolution. I am sure that no one in that discussion used any language that could by any possibility be rationally given the color that this reporter published. This reporter should be pierced through and through by the arrows of professional and public scorn. He should be made to trot out his druggist imformant, and said druggist should be made to trot out his doctors, and every one, if any, engaged in such nefarious practices should be overwhelmned by an avalanche of professional and public contempt and scorn. You cannot dip your pen in gall too concentrated to do justice to this vile article and its writer. Yours truly,

J. S. POYNER.

The above should be sufficient to fix upon this *Statesman* reporter either a willful perversion of facts or compel an acknowledgment that he was mistaken and has done the profession an injustice; and at the same time vindicate the delegate referred to and the entire medical profession. The young man reporter refuses to give the name of his druggist friend. If there is any one in Austin calling himself a physician who is guilty of such conduct as this alleged druggist imputes to him, his name ought to be published; but we don't believe there is a reputable physician in Austin who is guilty of such conduct.

